# Purification of Crude Fructo-Oligosaccharide Preparations Using Probiotic Bacteria for the Selective Fermentation of Monosaccharide Byproducts

**DOI:** 10.3389/fmicb.2020.620626

**Published:** 2021-01-26

**Authors:** Rong Fan, Jan Philipp Burghardt, Jinqing Huang, Tao Xiong, Peter Czermak

**Affiliations:** ^1^Institute of Bioprocess Engineering and Pharmaceutical Technology, University of Applied Sciences Mittelhessen, Giessen, Germany; ^2^Department of Bioresources, Fraunhofer Institute for Molecular Biology and Applied Ecology IME, Giessen, Germany; ^3^Faculty of Biology and Chemistry, Justus Liebig University, Giessen, Germany; ^4^State Key Laboratory of Food Science and Technology, Nanchang University, Nanchang, China

**Keywords:** probiotic microorganism, prebiotics, sugar metabolism, fructo-oligosaccharides purification, synbiotics

## Abstract

Probiotics are microbes that promote health when consumed in sufficient amounts. They are present in many fermented foods or can be provided directly as supplements. Probiotics utilize non-digestible prebiotic oligosaccharides for growth in the intestinal tract, contributing to a healthy microbiome. The oligosaccharides favored by probiotics are species-dependent, as shown by the selective utilization of substrates in mixed sugar solutions such as crude fructo-oligosaccharides (FOS). Enzymatically produced crude FOS preparations contain abundant monosaccharide byproducts, residual sucrose, and FOS varying in chain length. Here we investigated the metabolic profiles of four probiotic bacteria during the batch fermentation of crude FOS under controlled conditions. We found that *Bacillus subtilis* rapidly utilized most of the monosaccharides but little sucrose or FOS. We therefore tested the feasibility of a microbial fed-batch fermentation process for the purification of FOS from crude preparations, which increased the purity of FOS from 59.2 to 82.5% with a final concentration of 140 g·l^−1^. We also tested cell immobilization in alginate beads as a means to remove monosaccharides from crude FOS. This encapsulation concept establishes the basis for new synbiotic formulations that combine probiotic microbes and prebiotic oligosaccharides.

## Introduction

Probiotics are non-pathogenic microorganisms that live in the digestive tract of their host and promote health when consumed in sufficient quantities, for example, in fermented food such as yogurt (Food and Agriculture Organization of the United Nations, World Health Organization, 2006). They confer these benefits by regulating the growth of colonic bacteria, reducing serum cholesterol levels, and modulating inflammatory responses (Gibson and Wang, 1994; Aminlari et al., 2018; Liu et al., 2018). Typical probiotic microorganisms include lactic acid bacteria (LAB), bifidobacteria, and some yeasts. They span many genera, including *Bacillus*, *Bifidobacterium*, *Lactobacillus*, *Lactococcus*, *Leuconostoc*, and *Saccharomyces* (Fijan, 2014; Pandey et al., 2015). Probiotics are facultative anaerobic or microaerophilic microorganisms that ferment carbohydrates into lactic acid under anaerobic conditions, thus reducing the environmental pH and helping to inhibit pathogens in the intestine. They can also ferment non-digestible dietary fibers (prebiotics) into short-chain fatty acids in the colon (Markowiak and Śliżewska, 2017). In addition to organic acids, probiotic microbes can secrete various antimicrobial compounds such as hydrogen peroxide and bacteriocins (Reis et al., 2012). Probiotics therefore regulate the intestinal microbiome, inhibit the growth of pathogens, reduce inflammation, enhance the absorption of nutrients, and modulate host immune responses (Sánchez et al., 2010; Gu et al., 2015; Elshaghabee et al., 2017; Jäger et al., 2018; Monteiro et al., 2019).

The population of beneficial gut microbes can be increased directly by consuming probiotic foods or supplements, or indirectly by consuming prebiotic substrates that stimulate the growth of probiotic species. Probiotics are found naturally in fermented vegetables, milk and meat products (Xiong et al., 2013; Zhang et al., 2014). However, foods and supplements containing probiotics are vulnerable to inactivation by gastric acid, bile salts, and digestive enzymes. In contrast, non-digestible prebiotic oligosaccharides can reach the colon and modulate the beneficial intestinal microflora because they are stable in an acidic environment and are not absorbed in the upper gastrointestinal tract (Kaplan and Hutkins, 2003; Pandey et al., 2015; Singh et al., 2017; Figueiredo et al., 2020). Prebiotic oligosaccharides mainly consist of fructo-oligosaccharides (FOS), galacto-oligosaccharides (GOS), xylose-oligosaccharides (XOS) and inulin (Mutanda et al., 2014; Singh et al., 2017). A combination of probiotics and prebiotic substances (an approach known as synbiotics) can improve the survival and colonization rate of live microbial dietary supplements in the gastrointestinal tract (Pandey et al., 2015; Saneian et al., 2015).

In the traditional food sector, probiotic microorganisms are often used to produce fermented foods such as sauerkraut, cheese, yogurt, and preserved meat (Xiong et al., 2014, 2019). Furthermore, many strains of LAB have been isolated and used to produce lactic acid by industrial scale fermentation (Ye et al., 2014; Fan et al., 2017; Glaser and Venus, 2017; Olszewska-Widdrat et al., 2020). In recent decade, a novel application of probiotic microorganisms was reported in the literature. Probiotics such as *Saccharomyces cerevisiae* and *Bacillus coagulans* are used for the microbial purification of enzymatically produced crude FOS preparations, which also contain non-prebiotic monosaccharide byproducts and disaccharides (Nobre et al., 2016; Srivastava and Mishra, 2019; Fan et al., 2020a,b). The monosaccharides and disaccharides are removed to enhance the purity of FOS, and the monosaccharaides are converted into valuable byproducts such as organic acids and biomass, increasing the economic returns of the process.

Prebiotic oligosaccharides are mainly comprising of FOS, GOS, XOS, inulin, etc (Mutanda et al., 2014; Singh et al., 2017). FOS is a critical group of prebiotic carbohydrates featuring a sucrose molecule extended by a small number of fructose residues linked by β(2→1) glycosidic bonds (Sangeetha et al., 2005). The general formula is GF_n_ and specific examples include 1-kestose (GF_2_), nystose (GF_3_), and 1F-fructofuranosylnystose (GF_4_; Kovács et al., 2014; Ur Rehman et al., 2016). FOS can be obtained directly from natural sources like asparagus, garlic, chicory, onion and banana, or can be enzymatically produced from sucrose using fructosyltransferases (Jaime et al., 2001; Singh et al., 2017; Burghardt et al., 2019a,b). Enzymatically produced FOS are preferred by industry because the chain length can be controlled precisely by regulating the reaction conditions and duration. The crude product usually contains FOS, unreacted sucrose, and monosaccharide byproducts such as fructose and glucose. Our previous investigation revealed that *B. coagulans* can selectively consume small sugar molecules to increase the purity of FOS (Fan et al., 2020b). However, this preliminary study is still far from enough to be applied in practical process for FOS purification. This paper mainly focused on the establishment of a screening system to select an appropriate strain of *B. coagulans* for the integration of biomass production and FOS purification in terms of biomass accumulation, sporulation rate and preference for carbon sources in a mixture of monosaccharides, disaccharides, and oligosaccharides. Obviously, there are several key issues still left to be addressed: (1), despite variances in growth rate, biomass accumulation, and sporulation rate, different strains of *B. coagulans* had the similar manner in sugar metabolism. Besides the complete consumption of monosaccharides, they also consumed sucrose and short-chain FOS to some extent (40~50% for sucrose and 15~20% for FOS) during cultivation, leading to a waste of target products. (2) the purity of FOS in the final fermentation broth did not exceed 80% due to the residual sucrose. (3) the crude FOS solution must be diluted to a low concentration in the medium to avoid substrate inhibition in the simple batch process such that an appropriate enrichment approach was still needed to improve the technical feasibility for industrial application. Therefore, it is desirable to find microbes that selectively and efficiently convert monosaccharides with minimal consumption of FOS. Our questions are whether the selective consumption of monosaccharides can also be found in the metabolism of other probiotic microorganisms, and whether they could consume less FOS during their cultivation. And, which process design could be available to increase the FOS concentration in the final fermentation broth? This work aimed to answer these questions. However, there were very few studies reported in the literature concerning the selective consumption of carbon sources by probiotic microorganisms in a mixture of monosaccharides and oligosaccharides. Therefore, we compared the sugar consumption profiles of four different probiotic bacteria representing the genera *Lactobacillus* and *Bacillus* to identify a more appropriate candidate for the microbial purification of FOS with minimal loss of the target products. Here, we used the type strain of *B. coagulans* (DSM1) as a benchmark because its preference for monosaccharides in crude FOS has been well studied in the previous work. Moreover, we compared different bioprocesses (fed-batch fermentation and cell encapsulation) to confirm the technical feasibility of this approach for the microbial purification of FOS.

## Materials and Methods

### Microorganisms

Four probiotic bacteria were used in this study. *Bacillus coagulans* DSM 1 was obtained from the German Collection of Microorganisms and Cell Cultures [Deutsche Sammlung von Mikroorganismen und Zellkulturen GmbH (DSMZ), Braunschweig, Germany]. *Bacillus subtilis* YBJ was kindly provided by Huanong Hengqing Biotechnology, Co. Ltd., Gaoan, China. *Lactobacillus rhamnosus* NCU061001, and *Lactobacillus brevis* NCU002254 were isolated from traditional Chinese fermented vegetable Suansun and maintained by the State Key Laboratory of Food Science and Technology, Nanchang University, Nanchang, China. Verification of lactobacillus strains was assessed using ITS sequencing at Nanchang University, Nanchang, China (Guan et al., 2020).

### Culture Media

All bacteria were cultivated in a reduced-nutrient medium (3 g·l^−1^ yeast extract, 8 g·l^−1^ soybean peptone, 0.02 g·l^−1^ MnSO_4_, 1.5 g·l^−1^ KH_2_PO_4_, 1.5 g·l^−1^ K_2_HPO_4_, and pH 6.8). This concept is derived from DSMZ Medium 1.^1^ Meat extract and peptone from casein were replaced by yeast extract and soybean peptone with the equivalent nitrogen content to avoid the usage of animal-derived materials. The crude FOS preparation was added to this medium as a carbon source. A glucose yeast extract agar (GYEA) medium (5.0 g·l^−1^ casein peptone, 5.0 g·l^−1^ yeast extract, 2.0 g·l^−1^ glucose, 0.5 g·l^−1^ KH_2_PO_4_, 0.5 g·l^−1^ K_2_HPO_4_, 0.3 g·l^−1^ MgSO_4_, 0.01 g·l^−1^ MnSO_4_, 0.01 g·l^−1^ NaCl, and 15 g·l^−1^ agar) was used to determine the cell and spore counts. All components were sterilized at 121°C for 20 min except the sugar solution, which was filter-sterilized separately before mixing with other ingredients.

### Cultivation of Probiotic Bacteria in a Bioreactor

The probiotic bacteria were fermented in a 3-L bioreactor (Applikon Biotechnology, Delft, Netherlands) with a working volume of 1.5 L. Fermentations were carried out at 40°C with an aeration rate of 0.5 vvm. The pH was adjusted to 6.8 using 2 M NaOH.

The pre-culture was prepared in a 500-ml conical flask containing 100 ml medium. The inoculum was cultured for 14 h to reach the exponential phase. The fermentation bioreactor was inoculated with the pre-culture at an initial optical density at 600 nm (OD_600_) of 0.1 ± 10%. The OD_600_ was measured in triplicate by UV/Vis spectrophotometry (Eppendorf, Hamburg, Germany). An aliquot was diluted with physiological saline to ensure the OD value was <0.5. Cell growth was described by calculating the specific growth rate (*μ*) using Equation (1), where t_1_ and t_2_ refer to the start and end of each sampling interval. The highest value of μ during cultivation represented the growth rate in the exponential phase (μ_max_).

(1) $$\mu =\frac{\ln\frac{OD_{600,}{}_{t2}}{OD_{600,}{}_{t1}}}{t_{2}-t_{1}}\;$$

A fed-batch process was developed to increase the concentration and purity of FOS based on the batch fermentation data. The process started with a short batch fermentation phase, but as soon as the cells reached the last part of exponential growth, the fermentation was switched to fed-batch mode by continuously adding undiluted crude FOS solution with a glucose concentration of 148 g·l^−1^ at a constant flow rate of 20 ml·h^−1^.

The number of viable cells was determined in a plating assay and expressed as colony forming units (cfu). In each case, we prepared a 10-fold dilution series from the broth and streaked 50 μl of each sample on a GYEA plate. The number of colonies was counted after incubation at 40°C for 24–48 h. The arithmetic mean cell number in 1 ml of fermentation broth was calculated using the colony numbers of two consecutive decimal dilution levels according to Equation (2), where *N* is the arithmetic mean cell number in 1 ml of undiluted sample and 10^x^ is the dilution factor for the lowest evaluated dilution level. For example, when we used the colony numbers on the plates for 10^6^-fold and 10^7^-fold dilutions to calculate the cell number, the lowest evaluated dilution level was 10^6^. *V* is the volume of the sample streaked on each plate, *n*_x_ and *n*_x-1_ refer to the colony numbers on each plate, and *m*_x_ and *m*_x-1_ are the numbers of streaked plates at each dilution level.

(2) $$N=\frac{10^{x}}{V}\cdot \frac{\sum n_{x}+\sum n_{x+1}}{m_{x}+0.1m_{x+1}}$$

### Encapsulation of Probiotic Cells in Alginate Beads

Alginate beads were prepared by mixing 2 g sodium alginate in 100 ml culture medium 2 × stock solution (6 g·l^−1^ yeast extract, 16 g·l^−1^ soybean peptone, 0.04 g·l^−1^ MnSO_4_, 3 g·l^−1^ KH_2_PO_4_, 3 g·l^−1^ K_2_HPO_4_, and pH = 6.8) and sterilizing the mixture at 121°C for 20 min. We then mixed 50 ml of the cooled 2% sodium alginate medium with 0.5 ml pre-culture of probiotic bacterium and extruded the cell-containing sodium alginate solution through a 25G sterile cannula (inner diameter = 0.32 mm) in the form of droplets into 150 ml of 100 mM sterile CaCl_2_, while stirring at 100 rpm to form the beads. After 1 h, the hardened beads were rinsed with sterile sugar-free culture medium to remove free cells and CaCl_2_. The beads were then added to the diluted FOS solution at a 1:1 ratio, and incubated at 40°C for 24 h to evaluate the efficiency of FOS purification.

### FOS Synthesis

The commercial enzyme preparation Pectinex Ultra SP-L was used for the synthesis of FOS, with 600 g·l^−1^ sucrose as the substrate buffered with 0.1 M potassium phosphate (pH 5.5). The sucrose solution was mixed with 1% (v/v) enzyme preparation and incubated at 55°C on a heating plate stirring at 200 rpm for 24 h. The reaction was terminated by thermal enzyme deactivation at 80°C for 20 min.

### Analytical Method

The concentration of each component in the FOS solution was determined by ultra-high-performance liquid chromatography (UHPLC) as previously described (Fan et al., 2020b). Lactate concentrations were measured using a Biosen C line GP device (EKF-diagnostic, Barleben, Germany).

## Results

### Growth of Probiotic Bacteria in the FOS-Containing Medium

The crude FOS solution produced by the commercial enzyme preparation Pectinex Ultra SP-L contained 28.3 g·l^−1^ fructose, 148.3 g·l^−1^ glucose, 71.3 g·l^−1^ sucrose, 163.6 g·l^−1^ 1-kestose, 172.2 g·l^−1^ nystose, and 16.6 g·l^−1^ 1F-fructofuranosylnystose. This was comparable to the results of previous experiments under similar conditions (Aslan and Tanrıseven, 2007; Fan et al., 2020b). Given the high concentration of carbohydrates, this stock solution was diluted in the medium for the cultivation of probiotic bacteria with a glucose concentration of 20 g·l^−1^, thus avoiding any inhibitory effects.

**Figure 1:**
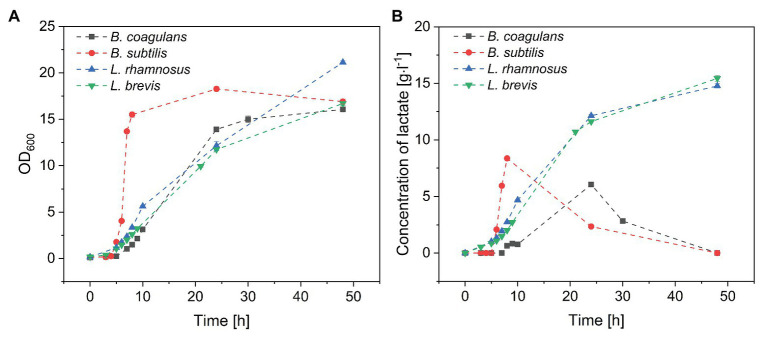
Batch fermentation of probiotic bacteria in medium containing crude fructo-oligosaccharides (FOS). **(A)** Growth curve and **(B)** lactate concentration during the fermentation. *T* = 40°C, pH = 6.8, and agitation = 0.5 vvm. Values are means ± error (*n* = 2).

**Figure 2:**
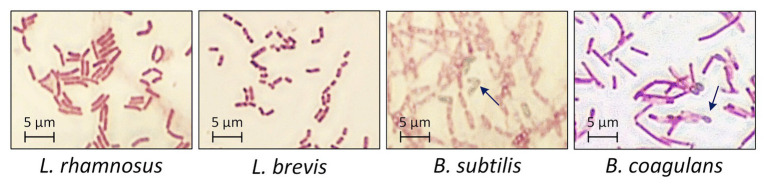
Morphology of probiotic bacteria after 48 h during the fermentation of crude FOS. *T* = 40°C, pH = 6.8, and agitation = 0.5 vvm. Arrows indicate the typical morphology of bacillus endospores.

The four probiotic species were cultivated for 48 h with an initial glucose concentration of 20 g·l^−1^ to compare their growth and biomass accumulation. As shown in Figures 1A,B*. subtilis* was the fastest-growing bacterium, reaching the exponential phase after a lag phase of 3 h (μ_max_ = 1.93), whereas *B. coagulans* had a longer lag phase (5 h) and a lower growth rate (μ_max_ = 0.71). However, the OD_600_ remained stable for both species of bacillus after reaching the maximum value. The lactobacilli grew more slowly than the bacilli (Table 1). However, the OD_600_ continued to increase throughout the fermentation for both *L. rhamnosus* and *L. brevis*. Notably, there were more viable lactobacilli than bacilli despite the lower OD_600_ of the lactobacillus cultures, reflecting differences in cell morphology (Figure 2). Accordingly, the relationship between OD_600_ and cell count was unique for each species.

**Table 1:** Cell count of probiotic bacteria after 24 h during the fermentation of crude FOS.

Species	μ_max_ [h^−1^]	Cell count [× 10^9^ cfu·ml^−1^]
*Bacillus coagulans*	0.71	2.14 ± 0.23
*Bacillus subtilis*	1.93	2.92 ± 0.03
*Lactobacillus rhamnosus*	0.41	4.66 ± 0.05
*Lactobacillus brevis*	0.52	4.31 ± 0.29

*T* = 40°C, pH = 6.8, and agitation = 0.5 vvm. Values are means ± error (*n* = 2).

Lactate, a metabolite of carbohydrates, was accumulated in the fermentation broth as a byproduct of carbohydrate utilization. As shown in Figure 1B, the concentration of lactate increased with increasing *B. subtilis* and *B. coagulans* biomass and reached maximum values by the end of the exponential phase. During the stationary phase, the concentration of lactate gradually declined to undetectable levels for both bacilli. In contrast, the lactate concentration increased throughout the fermentation for both *L. rhamnosus* and *L. brevis*, ultimately reaching ~15 g·l^−1^, which was consistent with the corresponding growth curves.

### Metabolism of Sugars in Crude FOS by Probiotic Bacteria

#### Metabolism of Monosaccharides

**Figure 3:**
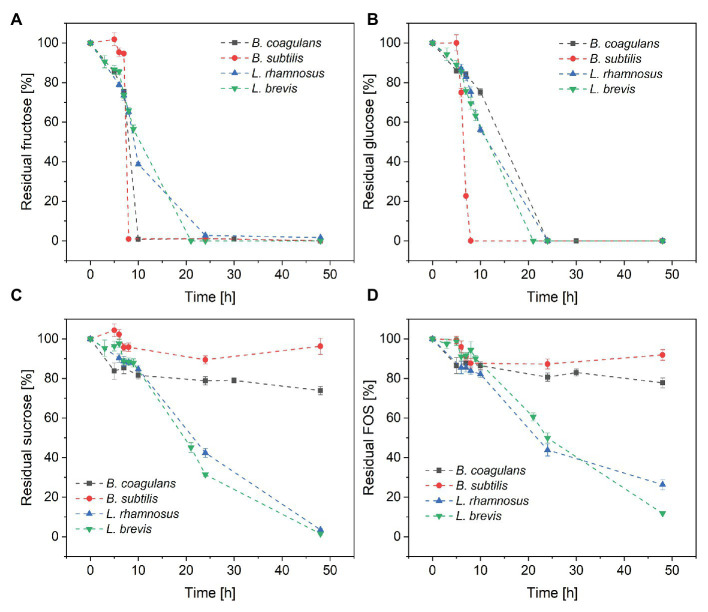
Metabolism of the sugar components in crude FOS by probiotic bacteria during a fermentation lasting 48 h: **(A)** fructose **(B)** glucose **(C)** sucrose, and **(D)** FOS. *T* = 40°C, pH = 6.8, and aeration = 0.5 vvm. Values are means ± error (*n* = 2).

Changes in sugar concentration during the cultivation of all four species of probiotic bacteria are shown in Figure 3. All four species completely consumed the fructose (Figure 3A) and glucose (Figure 3B) within 24 h, suggesting that monosaccharides were favored over sucrose and FOS. Furthermore, *B. subtilis* consumed all the glucose and fructose within 8 h, with a maximum glucose consumption rate of 7.5 g·l^−1^·h^−1^. In contrast, *B. coagulans* consumed all the fructose within 10 h but only ~25% of the glucose, suggesting that *B. coagulans* favors fructose over glucose. *Lactobacillus rhamnosus* and *Lactobacillus brevis* consumed both monosaccharides in a similar manner, such that the concentrations of fructose and glucose declined simultaneously to zero within 24 h.

#### Metabolism of Residual Sucrose

Although both bacilli were able to consume all the monosaccharides in the fermentation medium within ~25 h, only a small amount of sucrose was consumed by *B. coagulans* (<25%) and *B. subtilis* (<5%) after 48 h. In contrast, both of the lactobacilli were able to consume >95% of the sucrose by the end of the fermentation (Figure 3C). Furthermore, the lactobacilli metabolized the sucrose later than the monosaccharides, with ~50% of glucose and fructose consumed after 10 h but <20% of the sucrose.

#### Metabolism of FOS

The consumption of FOS mirrored the consumption of sucrose (Figure 3D). Only small amounts of FOS were consumed by *B. coagulans* (<23%) and *B. subtilis* (<9%) after 48 h, whereas a much larger amount was consumed by *L. rhamnosus* (~90%) and *L. brevis* (~75%). As with sucrose, most of the consumption of FOS by the lactobacilli was detected after the depletion of monosaccharides.

### Purification of FOS Using *Bacillus subtilis*

#### Encapsulation of *Bacillus subtilis* in Alginate Beads

**Figure 4:**
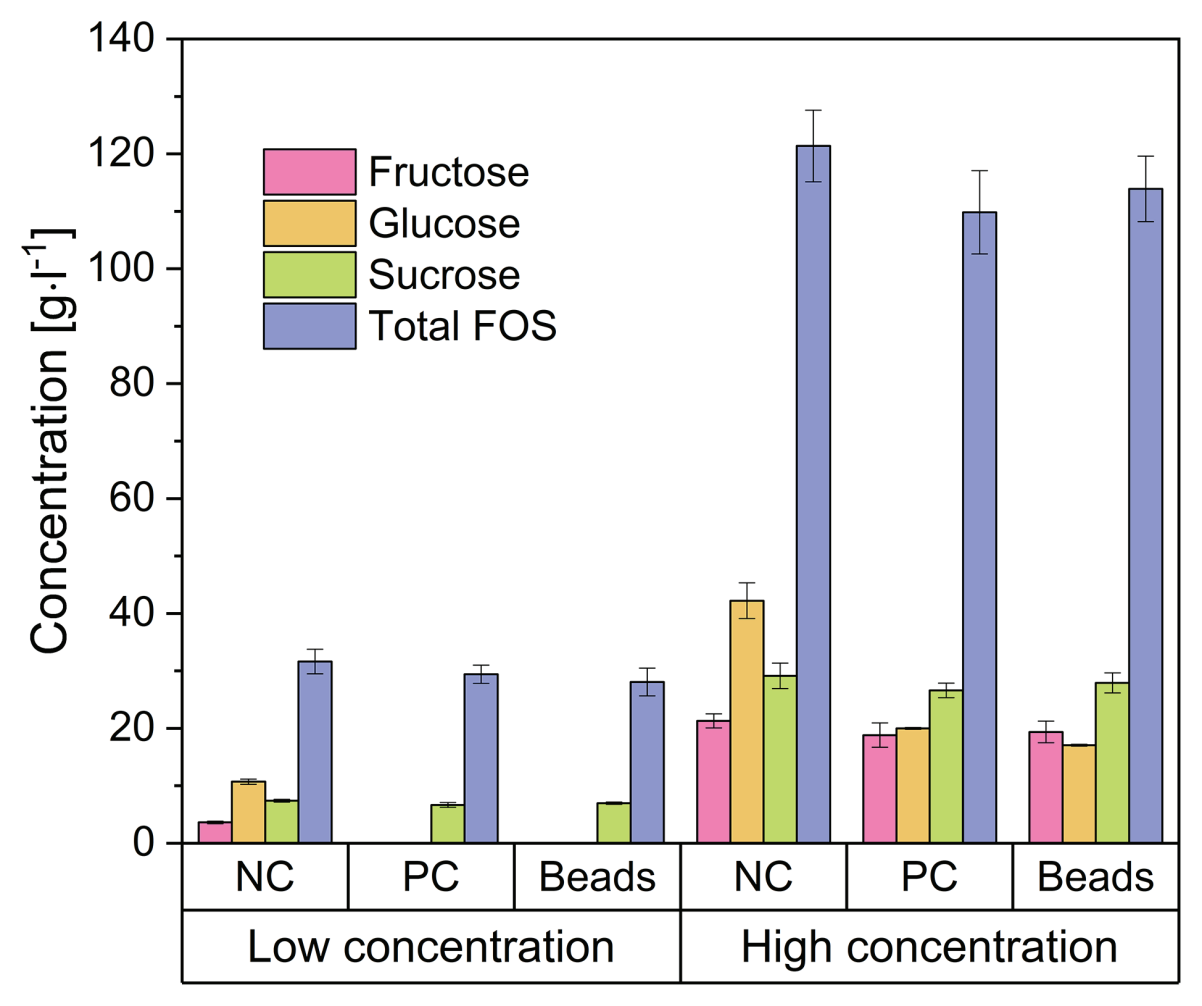
Concentration of the components of a FOS solution mixed with encapsulated *Bacillus subtilis*. The low and high concentrations refer to FOS solutions containing 20 and 80 g·l^−1^ of glucose, respectively, which was reduced to 50% by adding an equal volume of alginate beads. Negative control (NC) = 25 ml FOS solution + 25 g cell-free alginate beads. Positive control (PC) = 25 ml FOS solution + 24.5 ml 2 × stock solution of cell free medium + 0.5 ml *B. subtilis* pre-culture. Beads = 25 ml diluted FOS solution + 25 g alginate beads with immobilized *B. subtilis*. The concentration was determined after incubation at 40°C for 24 h. Values are means ± error (*n* = 2).

We encapsulated *B. subtilis* in alginate beads and added them to FOS solutions with glucose concentrations of 20 and 80 g·l^−1^. Figure 4 shows the concentrations of the major sugar components in the solution after 24 h. The addition of the beads doubled the volume of the solution and thus immediately reduced the concentration of all components by 50%, and these are the values represented by the negative control (NC). We found that the concentration of each sugar declined when treated with the encapsulated cells, and the profile was similar in the positive control (PC) treatment with free cells. The monosaccharides were completely consumed in the crude FOS preparation containing 20 g·l^−1^ glucose whereas there was little change in the concentrations of sucrose or FOS. In the crude FOS preparation containing 80 g·l^−1^ glucose, half the glucose was consumed but there was little change in the concentrations of the other components (including fructose). The pH also decreased (from ~6.8 to 4.2) during the cultivation, causing the inhibition of cell growth and metabolism by the acidic environment.

#### Fed-Batch Fermentation With Free Cells for the Purification of FOS

**Figure 5:**
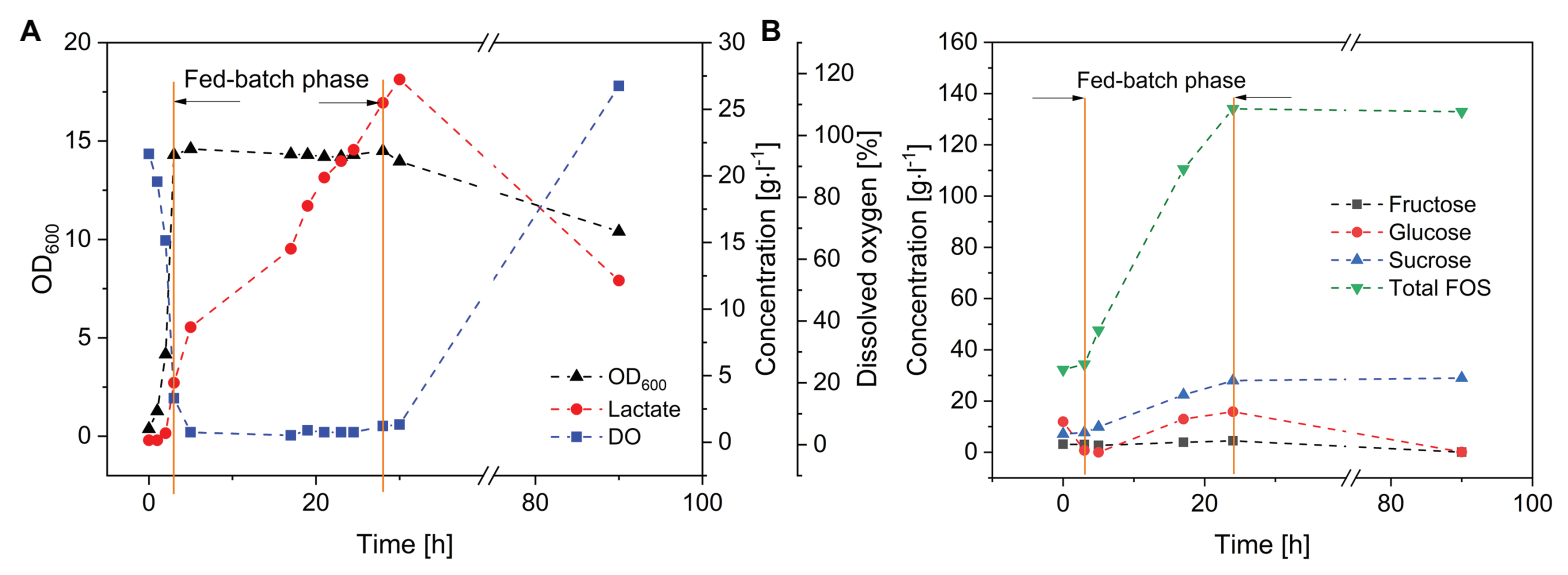
Fed-batch fermentation of *B. subtilis* in a 3-L stirred-tank bioreactor. Feeding rate of FOS solution = 20 ml·h^−1^, *T* = 40°C, pH = 6.8, and aeration = 0.5 vvm. **(A)** *Bacillus subtilis* growth kinetics and product formation, and **(B)** purification of FOS during the fermentation.

Finally, we carried out fed-batch fermentation for the purification and enrichment of FOS (Figure 5). A short batch phase with a low glucose concentration (set at 10 g·l^−1^ using a diluted FOS stock solution) was followed by the fed-batch phase in which the crude FOS preparation was supplied to avoid substrate inhibition. The fed-batch phase began when the number of viable cells was sufficient. During the 3-h batch fermentation, the monosaccharides were rapidly consumed, whereas the concentrations of sucrose and FOS remained stable. The glucose feeding rate was set at 3 g·l^−1^·h^−1^, which is lower than the maximum glucose consumption rate of *B. subtilis*. The cells were able to consume glucose and fructose simultaneously. Due to the highly selective consumption of monosaccharides, FOS was enriched in the fermentation broth while fructose and glucose were depleted. At the end of the process, the purity of FOS had increased from 59.2% in the crude preparation to 82.5% in the fermentation broth, with a final concentration of 140 g·l^−1^.

## Discussion

The growth of probiotic microorganisms on single carbon sources such as glucose, sucrose or FOS has been widely investigated and the metabolic profiles are well understood. However, very few studies were reported concerning the selective consumption of carbon sources by probiotic microorganisms in a mixture of monosaccharides and oligosaccharides. To our knowledge, it has been confirmed that bifidobacteria and some species of lactobacillus can utilize FOS (Rossi et al., 2005). Different species even have their own preference on certain oligosaccharides (Tabasco et al., 2014). This work aimed to select an appropriate probiotic microorganism that can preferentially consume monosaccharides with a minimum loss of FOS in the mixed sugar system. Our results confirmed that one or more components in a crude FOS preparation could support the growth of probiotic bacteria, but that different bacterial species show different metabolic preferences. One key difference between the bacilli and lactobacilli was that more diverse sugar components were available to the latter, contributing to their higher cell count (which is a favorable property for probiotic formulations). However, the bacilli were able to form endospores after cultivation for 48 h, offering greater protection against harsh conditions in the digestive tract, such as bile salts and gastric acid (Gu et al., 2015). This property could compensate for the lower cell count of the two species of bacilli.

The growth of probiotic bacteria can be determined not only directly by measuring biomass accumulation, but also indirectly by measuring the production of lactate. During the fermentations with *B. coagulans* and *B. subtilis*, the lactate concentration peaked during the exponential phase, followed by a decline during the stationary phase. Lactate is produced from the fermentable carbohydrates under anaerobic conditions, so the lactate peak suggested the complete consumption of available sugar-based carbon sources followed by the utilization of lactate as an alternative. In contrast, the lactate concentration kept increasing during the growth of *L. rhamnosus* and *L. brevis*, suggesting that sufficient substrate remained to allow the conversion of carbohydrates into lactate throughout the fermentation. Sugar analysis could provide more insight into the differential utilization of carbohydrates by bacilli and lactobacilli.

The metabolism of sugars occurs *via* different pathways depending on the chain length. The utilization of monosaccharides is a fundamental aspect of energy metabolism in all living organisms. Glucose and fructose are imported into cells and phosphorylated, allowing their utilization *via* glycolysis. The metabolism of disaccharides and larger oligosaccharides is more complex, with at least three pathways existing in lactobacilli: first, the sugars can be converted into monosaccharides by extracellular glycoside hydrolases (such as invertase) and utilized as discussed above; second, the sugars can be taken up and phosphorylated mediated by the sucrose phosphotransferase system (Pts1BCA) or oligosaccharide transporter (MsmEFGK) before hydrolysis by an intracellular phosphofructo-furanosidase; and third, they can be taken up and converted into glucose 1-phosphate and a fructose/fructan *via* phosphorolysis (Kaplan and Hutkins, 2003; Reid and Abratt, 2005; Saulnier et al., 2007; Gänzle and Follador, 2012; Awad et al., 2013). Two or more of the above pathways operate in most lactobacilli, explaining why numerous strains of lactobacilli grow on FOS and confer probiotic effects (Endo et al., 2016). However, the presence of glucose may inhibit or repress the enzymes and other proteins required for sucrose/FOS hydrolysis and transport. For example, glucose reversibly interferes with the transcription and translation of invertase (Elorza et al., 1977). This explains why the lactobacilli in our study started to extensively metabolize FOS only after the depletion of monosaccharides. Other studies have provided evidence for irreversible inhibition by hexoses. For example, *Lactobacillus paracasei* growing on glucose was not able to import FOS, suggesting the absence of a membrane-based transport system for FOS in the presence of glucose (Kaplan and Hutkins, 2003). This may explain why the two bacilli we tested consumed little sucrose or FOS even after the depletion of monosaccharides.

More investigations at the level of molecular biology and biochemistry are still needed to reveal the difference between the metabolic behaviors of lactobacilli and bacilli in the mixed sugar system. Nevertheless, the selective consumption of monosaccharides by the bacilli is advantageous for the purification and enrichment of FOS. *Bacillus subtilis* appears more suitable than *B. coagulans* for this application because it has a higher growth rate, higher glucose consumption rate, and lower loss of FOS. We therefore used *B. subtilis* to develop a process for the purification and enrichment of FOS based on cell encapsulation within alginate beads. We found that the encapsulated cells could remove monosaccharides with the same efficiency as free cells but were easier to remove from the medium, thus simplifying and reducing the costs of downstream processing. Furthermore, the beads can be used in fixed-bed reactors for the continuous purification of FOS solution. In addition, many studies reported that alginate-based capsules can also protect probiotic microbes against the effects of gastric acid, extending their survival in the digestive tract (Chávarri et al., 2010; Mei et al., 2014; Petraitytė and Šipailienė, 2019). Because the beads in this study contained viable probiotic cells as well as purified prebiotic FOS, the encapsulation of probiotic microorganisms is also a promising concept for the preparation of novel synbiotic formulations, increasing the value of the entire process.

The efficiency of the encapsulated cells for the purification of FOS was limited by factors such as substrate inhibition and acidic metabolites in the fermentation broth. We therefore developed a fed-batch fermentation process to increase the final concentration of FOS. Yeasts such as *Saccharomyces cerevisiae* and *Kluyveromyces lactis* have previously been used to remove monosaccharides and disaccharides from crude preparations of prebiotic oligosaccharides (Nobre et al., 2016; Castro et al., 2019) resulting in FOS concentrations of up to 182.7 g·l^−1^ with a purity of 98.2% (Sheu et al., 2013). Microbial treatment is therefore a feasible approach for the purification and enrichment of enzymatically synthesized oligosaccharides.

## Conclusion

In this study, we characterized the different metabolic behaviors of probiotic bacilli and lactobacilli in a mixed sugar system. The lactobacilli were able to utilize all the components of a crude FOS preparation whereas the bacilli favored the monosaccharides and were unable to utilize sucrose or FOS to any significant extent. In particular, *B. subtilis* quickly consumed the monosaccharides without any obvious reduction in the concentration of FOS, making this species suitable as a microbial tool for FOS purification. In a fed-batch fermentation process, we increased the total concentration of FOS to three times the value achieved in a batch process, with a purity of 82.5%. Furthermore, *B. subtilis* cells immobilized in alginate beads provided an alternative strategy to produce synbiotic preparations in addition to the purification of FOS.

## Data Availability Statement

The original contributions presented in the study are included in the article/supplementary material and further inquiries can be directed to the corresponding authors.

## Author Contributions

RF designed and carried out the experiments, analyzed the results, wrote the manuscript, and takes responsibility for the interpretation of all the data. JB developed and conducted the UHPLC analysis. JH and TX helped with the strain isolation and screening. PC led the research program and developed the experimental approach. All authors contributed to the article and approved the submitted version.

### Conflict of Interest

The authors declare that the research was conducted in the absence of any commercial or financial relationships that could be construed as a potential conflict of interest.

## Footnotes

^1^https://www.dsmz.de/microorganisms/medium/pdf/DSMZ_Medium1.pdf

## Abbreviations

cfu: colony forming unit
DSMZ: Deutsche Sammlung von Mikroorganismen und Zellkulturen GmbH (German Collection of Microorganisms and Cell Cultures)
FOS: fructo-oligosaccharides
GF_2_: 1-kestose
GF_3_: nystose
GF_4_: fructofranosyl nystose
GOS: galacto-oligosaccharide
GYEA: glucose yeast extract agar
LAB: lactic acid bacteria
OD_600_: optical density at *λ* = 600 nm
NC: negative control
PC: positive control
UHPLC ultra-high-performance liquid chromatography
XOS: xylose-oligosaccharide
